# Cohesin regulation of genome organization in mature granule neurons in the mouse cerebellum

**DOI:** 10.1186/s13072-025-00625-2

**Published:** 2025-09-26

**Authors:** Omar A. Payán Parra, Ziyu Zhao, Tomoko Yamada, Yue Yang

**Affiliations:** 1 Department of Neurobiology, Northwestern University, Evanston, IL USA; 2 Program in Interdisciplinary Biological Sciences, Northwestern University, Evanston, IL USA

## Abstract

**Background:**

Proper control of gene expression is important for the development and functions of neurons in the brain. The three-dimensional organization of the genome facilitates gene expression by regulating interactions between gene promoters and their enhancers. Notably, the cohesin complex drives genome folding through loop extrusion, thereby increasing promoter-enhancer interactions. Although cohesin's roles have been well-characterized in proliferating cells and cultured developing neurons, its functions in nuclear organization and gene transcription in mature mammalian brain neurons in vivo remain incompletely understood.

**Results:**

To investigate cohesin's functions in the brain, we induced the conditional knockout of the core cohesin subunit RAD21 specifically in cerebellar granule neurons during late development or in adulthood. We then performed RNA-seq and Hi-C approaches to determine the effects of RAD21 depletion on gene expression and 3D genome organization. We found that cohesin was required for the expression of genes that become active in mature granule neurons, and this was linked to its functions in increasing local genomic interactions that bring target gene promoters into spatial proximity with their enhancers. Moreover, for target genes with distal intergenic enhancers, cohesin also maintained those intergenic enhancers within the transcriptionally active A compartment.

**Conclusions:**

Our results reveal the essential functions of cohesin in gene transcription by regulating genome folding across multiple length scales in cerebellar granule neurons. Its roles in orchestrating both local and compartment-level genomic interactions highlight the additional layers of regulation for genes selectively expressed in mature post-mitotic neurons in vivo.

**Supplementary Information:**

The online version contains supplementary material available at 10.1186/s13072-025-00625-2.

## Background

Gene expression is coordinated throughout neuronal development and adulthood to ensure the proper formation and function of the brain [1–3]. The three-dimensional (3D) organization of the genome, including its folding within local structural domains and its associations over longer distances with nuclear compartments, may play important roles in regulating these gene expression patterns [4, 5]. Human genetics studies have implicated genes encoding genome architectural proteins in neurodevelopmental and psychiatric disorders [6, 7]. However, how these proteins regulate the genome organization of post-mitotic neurons in the brain remains incompletely understood.

At a local level of genome organization, regulatory enhancers form loops with gene promoters located up to several hundred kilobases (Kb) away to regulate transcription [8]. The association between active enhancers and gene promoters recruits and increases the local concentration of transcription factors and coactivators at promoters, thereby facilitating gene transcription [9, 10]. Cohesin is a key regulator of local genome organization and may stimulate promoter-enhancer contacts through its loop extrusion functions [11]. However, these interactions are largely confined within topologically associating domains (TADs), which span up to a few megabases (Mb) [12–14]. TAD boundaries are enriched for the CCCTC-binding factor (CTCF), which acts as a barrier for cohesin-mediated loop extrusion [15, 16]. At the chromosomal level beyond TADs, the genome is further separated into a transcriptionally active A compartment and a repressive B compartment [17]. These compartments reflect the spatial partitioning of euchromatin and heterochromatin within the nucleus [18–20]. Notably, cohesin appears to suppress inter-TAD compartment interactions in proliferating cells [13, 21].

Although cohesin promotes local genomic interactions and suppresses longer distance interactions, it appears to have limited effects on gene expression in proliferating cells [14]. Instead, deletion of cohesin subunits in post-mitotic neurons leads to dysregulated transcription and impaired dendrite and synaptic maturation [22, 23], indicating specialized roles for cohesin in the brain. Indeed, mice with chronically reduced cohesin levels exhibit increased anxiety-related behaviors [23]. In adult mice, cohesin is required for the induction of activity-dependent gene expression programs that are required for long-term memory [24]. Cohesin also organizes chromatin loops that activate regeneration-associated transcriptional programs after sciatic nerve injury [25]. However, beyond its roles in the activation gene expression in response to extrinsic and cell-intrinsic cues, it remains unknown whether cohesin is also necessary for maintaining these gene expression programs in mature neurons in the adult brain.

In this study, we investigated the roles of cohesin in gene transcription and genome organization in mature cerebellar granule neurons in vivo. Using the CRISPR-Cas9 system, we induced the conditional knockout of the core cohesin subunit RAD21 specifically in mature granule neurons, bypassing earlier developmental stages. We then biochemically profiled RAD21 conditional knockout or control granule neurons using fluorescence-activated nuclei sorting (FANS) followed by RNA-seq or Hi-C analyses. These data were integrated with our previous datasets where RAD21 was silenced later in adulthood [24]. We found that RAD21 depletion dysregulated hundreds of genes, including many that were selectively expressed in mature granule neurons and were bound by RAD21 at their promoters. Cohesin facilitated local interactions between target gene promoters and their enhancers, and for a subset of genes, also maintained the association of their distal intergenic enhancers with the active A compartment. Together, our findings show that cohesin organizes the genome at multiple length scales to control gene transcription in mature cerebellar granule neurons in vivo.

## Methods

### Animals

Homozygous LSL-Cas9-EGFP (#026175, Jackson Laboratory) mice were bred with GABA(A)Rα6-Cre driver mice [26] for in vivo electroporation. Both male and female mice were used.

### Antibodies

Antibodies to RAD21 (Abcam, ab992), CTCF (Millipore, 07-729), NeuN (Abcam, ab177487), and mCherry (Abcam, ab205402) for immunohistochemistry or FANS experiments were purchased.

### Plasmid DNA

sgRNA was cloned into the MLM3636 vector (Addgene #43860) using primers containing the target sequence: *Rad21*: ATGCTTCATTACAGTCTGCG (chr15: 51978075-51978094) and control: CGAGAGCAGGTCTGAAACCC (chr12: 8304558083045599) and GGAGGGTAGATTAGATTAGC (chr12: 8304548583045504). mCherry-NLS (addgene #58476) was subcloned into the pCAG vector to generate pCAG-mCherry-NLS.

### In vivo electroporation

In vivo electroporation of Gabra6-Cre; LSL-Cas9-EGFP mice conditionally expressing wild type Cas9 in mature granule neurons (Cas9-GC) was performed as described [27, 28]. The indicated plasmids were injected into the cerebellum of P6 mouse pups and then subjected to five 50 ms electrical pulses of 135 mV with 950 ms intervals. Electroporated pups were returned to moms and subjected to biochemical or immunohistochemical analyses at the indicated days after electroporation.

### Statistics

Statistical analyses were performed using R. Normality of data was assessed using the Shapiro–Wilk test. The t-test was used for normal distributions. For skewed distributions, the sign test (paired data) was used. Comparisons of samples across multiple groups were performed using Mood’s median test (unpaired data) followed by Benjamini–Hochberg correction to compare every mean with a control mean for skewed distributions.

### ChIP-seq

ChIP-seq assays were performed as described with modifications [27, 28]. The cerebellum from postnatal day 6 or 22 mice was fixed with 1.1% formaldehyde solution. Immunoprecipitation was performed in RIPA buffer (10 mM Tris–HCl pH 8.0, 140 mM NaCl, 0.1% SDS, 1% Triton-X, 0.1% DOC, 1 mM EDTA, 0.5 mM EGTA) using the indicated antibodies with Dynabeads protein G (Thermo Fisher Scientific) or Protein G Sepharose (GE Healthcare).

The lysate was mixed with an antibody against RAD21 or CTCF overnight at 4C. BSA-coated beads (Dynabeads protein G for RAD21; Protein G Sepharose for CTCF) was added and incubated at 4C for 1 h. After extensive washes of beads with RIPA buffer three times, high salt RIPA buffer (10 mM Tris–HCl pH 8.0, 500 mM NaCl, 0.1% SDS, 1% Triton-X, 0.1% DOC, 1 mM EDTA, 0.5 mM EGTA) three times, and TE three times, DNA fragments were eluted in elution buffer (100 mM NaHCO3, 1% SDS, 10 mM DTT at room temperature for Sepharose beads; 10 mM Tris–HCl pH 8.0, 350 mM NaCl, 1% SDS, 0.1 mM EDTA at 65C for Dynabeads) for 30 min, treated with proteinase K for 1 h at 37C, and de-crosslinked at 65C overnight. DNA fragments were purified with a PCR purification kit (Qiagen). Libraries were prepared using a NEBNext Ultra™ II DNA Library Prep Kit for Illumina (New England Biolabs) as per the manufacturer’s instructions.

All libraries were sequenced on an Illumina NextSeq 500 platform to obtain 36-37 bp paired-end reads. Two to four biological ChIP-seq replicates were performed in all experiments.

### FANS

The cerebellum of mouse pups electroporated with the pCAG-mCherry-NLS plasmid together with a vector encoding U6-sgRNA targeting *Rad21* or control sequences was dissected 28 days after electroporation under a stereomicroscope (Olympus SZX16) to assess mCherry expression. The cerebellar vermis was fixed with crosslink solution (1% PFA in PBS) by homogenizing with a glass dounce homogenizer and incubated for 10 min at room temperature. Crosslinking reaction was quenched by 125 mM glycine solution for 5 min at room temperature. The cell pellet was washed twice with BSA/PBS solution (0.3% BSA, 0.1% Triton-X in PBS) and stored at -80C.

To isolate nuclei, the frozen pellet was thawed on ice, homogenized with lysis buffer (10 mM Tris–HCl pH 8, 10 mM NaCl, 0.2% Triton-X) and incubated on ice for 10 min. The lysate was pelleted by centrifugation at 700 g for 5 min at 4C and washed with lysis buffer once. The pellet was resuspended in BSA/PBS solution and filtered using a 50 µm filter (CellTrics). Filtered nuclei were incubated with antibodies against mCherry (1:1000) and NeuN (1:500) for 1 h at 4C with rotation. Nuclei were washed with BSA/PBS solution twice and stained with secondary antibodies (anti-chicken Alexa 488, 1:250, Invitrogen, A11039; anti-rabbit Alexa 647, 1:250, Abcam, ab150075) for 30 min at 4C with rotation, followed by washing with BSA/PBS solution twice. Stained nuclei were resuspended in BSA/PBS solution and filtered using a 50 µm filter prior to flow cytometry. For RNA-seq sample preparation, 0.4 unit/μl RNase inhibitor (Promega, N2515) was added to all solutions except for the wash buffer. mCherry^+^/NeuN^+^ nuclei were enriched with a SH800S cell sorter (Sony) using a 100 µm nozzle sorting chip and 488/638 nm lasers. Nuclei were first sorted using the ultra-yield mode and then re-sorted using the normal mode.

### FANS-RNAseq

For RNA-seq following FANS, sorted cells were frozen and stored at − 80C. ~ 2000 sorted nuclei from single or multiple animals were thawed on ice and reverse-crosslinked with 100 µL of RIP buffer (100 mM Tris–HCl pH 8.0, 10 mM EDTA, 1% SDS, 1 μl of RNase inhibitor (Promega, N2515), 1 µl of Proteinase K (New England Biolab)) for 1 h at 65C. Nuclear RNA was then purified using a RNeasy micro kit (Qiagen) according to the manufacturer’s instructions.

Half the amount of purified RNA from FANS-isolated granule neurons was treated with a NEBNext rRNA Depletion Kit (New England Biolabs). RNA-seq was performed using libraries prepared with a NEBNext Ultra™ Directional RNA Library Prep Kit for Illumina (New England Biolabs).

All libraries were sequenced on the Illumina NextSeq 550 platform to obtain 37 bp paired-end reads. Two to three biological RNA-seq replicates were performed for each condition.

### FANS-HiC

For Hi-C following FANS, ~ 2600 to 5500 cells were sorted into PBS and lysis buffer at four times the volume was added immediately. Hi-C was performed as described with modifications [13, 24]. Nuclei were pelleted and resuspended with 0.5% SDS and incubated at 62C for 10 min for permeabilization. Permeabilization was quenched by adding Triton X-100. The nuclei were treated with MboI (New England Biolabs) for 2–3 h, followed by biotin fill-in reaction using biotin-14-dATP (Thermo Fisher Scientific) with Klenow (New England Biolabs). Proximity ligation was performed at room temperature overnight using T4 ligase (New England Biolabs). To remove biotin from non-ligated ends, nuclei were treated with T4 DNA polymerase (New England Biolabs) in the presence of dGTP at 12C for 2 h. Nuclei were spun down at 700 g for 5 min and the supernatant was discarded. The nuclei were resuspended in nuclei lysis buffer (10 mM Tris–HCl pH 8.0, 350 mM NaCl, 0.1 mM EDTA, 1% SDS) and treated with 2 μg RNase A (Ambion) for 1 h at 37C, followed by 20 μg proteinase K overnight at 65C. The nuclei were sonicated (Bioruptor Pico, diagenode) until the size of DNA fragments was 300-700 bp, assessed by bioanalyzer. DNA fragments were purified by PCR purification kit (Qiagen). Biotin pull-down of ligated DNA was performed using MyOne Streptavidin T1 beads (Thermo Fisher Scientific). Hi-C libraries were generated from the beads-bound ligated DNA fragments using the NEBNext Ultra™ II DNA Library Prep Kit for Illumina (New England Biolabs) and sequenced on the Illumina NextSeq 550 platform to obtain 37 bp paired-end reads. Two biological Hi-C replicates were performed for each condition.

### Immunohistochemistry

The cerebellum of mouse pups electoporated with the pCAG-mCherry-NLS plasmid together with a vector encoding U6-sgRNA targeting *Rad21* or control sequences were dissected at day 14 or 28 after electroporation under a stereomicroscope (Olympus SZX16) and fixed with 4% PFA and 4% sucrose. Sagittal cryosections (12 µm thickness) were prepared using a cryostat (Epredia Cryostar NX70) and cerebellar sections containing the vermis area were used for analyses. Sections were treated with sodium citrate solution (10 mM sodium citrate, pH 6.0) for 10 min at 70C to denature the conditionally expressed EGFP. After cooling down to room temperature, sections were incubated with blocking buffer for 1 h at room temperature followed by an antibody against RAD21 (1:1000) at 4C overnight. Sections were then washed three times with PBS and labeled with a secondary antibody (anti-rabbit Alexa488, 1:250, Thermo Fisher Scientific, A11034) for 1 h at room temperature. Sections were then washed three times with PBS and were stained with the Hoechst dye. After washing with PBS, sections were mounted with Fluoromount-G mounting medium (Southern Biotech). Images were acquired at the Biological Imaging Facility on a confocal laser scanning microscope (Leica Microsystems, TCS SP8 confocal) with a 63 × objective lens (NA 1.40). A stack of 0.3 µm thick optical sections was acquired for each field of view in the UV, green, and red channels. Two biological replicates were performed for each condition.

### DNA-FISH combined with immunohistochemistry

DNA-FISH were performed as described [24] with modifications. Cerebellar sections were treated with sodium citrate solution as described above. Sections were then labeled with a mCherry antibody (1:1000) and a secondary antibody (anti-chicken Alexa488, 1:250, Thermo Fisher Scientific, A11039). After extensive washing with PBS, sections were fixed in 4% PFA/PBS for 10 min at room temperature. Sections were then washed with PBS for 5 min twice and with 2xSSC once for 5 min, and treated with ice-cold HCl solution (0.1 M HCl, 0.7% Triton-X) for 15 min at room temperature. After washing with 2xSSC, genomic DNA was denatured by incubating glass plates with prehybridization buffers containing 2 × SSC/70% formamide for 2.5 min and 2 × SSC/50% formamide for 1 min at 73C. All probes were prepared from BAC clones (BACPAC Resources Center) including RP24-397M16 (*Dpf3* promoter locus) and RP24-88C18 (*Dpf3* enhancer locus) by nick translation and chemical coupling using an Alexa Fluor succinimidyl ester (AF647 for *Dpf3* promoter; AF555 for *Dpf3* enhancer) (Thermo Fisher Scientific). The hybridization solution contained ~ 70 to 80 ng of each labeled probe, 6 μg of mouse Cot-1 DNA (Thermo Fisher Scientific), and 10 μg of sheared salmon-sperm DNA (Thermo Fisher Scientific) in hybridization buffer (10% dextran sulfate, 50% formamide, 2xSSC). The probe mixtures were denatured at 73C for 5 min before use. Denatured sections and probes were sealed and incubated at 37C overnight. Coverslips were then removed and sections were washed once in 2 × SSC and 50% formamide solution for 15 min at 45C and three times in 2 × SSC for 5 min at 45C with gentle agitation. Sections were washed once with PBS, stained with the Hoechst dye, and prepared for imaging as described above. Two biological replicates were performed in all experiments.

### DNA FISH and immunohistochemical analyses

RAD21 fluorescence images were subjected to a 3D Gaussian Blur to reduce noise, and fluorescence levels in mCherry-positive electroporated cells were calculated and normalized to the mean levels in three nearby non-electroporated cells.

The 3D distance between genomic loci was analyzed using custom macros in ImageJ. DNA FISH signals were subjected to a 3D Gaussian filter followed by 3D spot segmentation. The Euclidian distance between two DNA FISH probes was then calculated.

### Hi-C analyses

Hi-C reads were aligned to the mm10 reference genome with Bowtie2 and HiC-Pro as described [29]. Uniquely mapped paired-end reads were assigned to MboI restriction fragments and valid pairs with a minimum genomic distance of 1 Kb were filtered for PCR duplicates. Interaction matrices were normalized using Knight-Ruiz (KR) matrix balancing and visualized using Juicebox [30]. To increase resolution, Hi-C datasets from P22, P34, and P56 control cerebellum including from this study and [24, 31] were combined and designated as adult cerebellum. Local gene body interactions were calculated using the mean normalized interactions between all 5 Kb bins spanning each transcript.

A/B compartment scores were derived from the first eigenvector of the Hi-C correlation matrix [13] using 25 Kb or 500 Kb bins. Genomic loci enriched for the active histone modification H3K27ac and de-enriched for the repressive histone modification H3K9me3 were assigned a positive eigenvector and designated as the active A compartment, while genomic loci with a negative eigenvector were designated as the repressive B compartment. To identify genomic features associated with compartment changes, A/B scores for all A compartment bins at 25 Kb resolution were curated and the changes in A/B score following RAD21 depletion were calculated. Bins were then ranked by the magnitude of score change and grouped into sets of 500. Within each group, the change in expression for nearest protein-coding gene (log₂ CPM > 0), the fraction of bins overlapping with gene bodies, or the distance to the nearest intergenic ChIP-seq peak non-overlapping with gene bodies or promoter regions extending 500 bp upstream of TSSs, was computed.

### RNA-seq analyses

For bulk RNA-seq analyses, sequenced reads were aligned to the mm10 reference genome with HISAT2 using the public server at https://usegalaxy.org/ and normalized by library size. Gene annotations were derived using GENCODE version M11. Differential gene expression analyses were performed using pair-wise negative binomial tests with edgeR [32] and the false discovery rate (FDR) was calculated for all genes. Gene ontology analyses of differentially expressed genes were performed using DAVID [33] Knowledgebase v2025_1 with a background of all expressed genes in both the P34 and P56 RNA-seq datasets.

### ChIP-seq analyses

ChIP-seq reads were aligned to the mm10 reference genome with Bowtie2 using the public server at https://usegalaxy.org/ and normalized by library size. Peak calling was performed using MACS2 [34]. H3K27ac peaks > 2 Kb distal to H3K4me3-bound TSSs were considered as enhancers.

ChIP-seq read density at promoters was calculated across merged H3K27ac peaks from P6 and P22 and that overlapped with H3K4me3-bound TSSs. To identify intergenic enhancers that were positioned nearby genes, only intergenic regions immediately flanking a gene, with no intervening protein-coding genes, were considered.

## Results

### RAD21 is recruited to genes to activate their expression in mature granule neurons

**Fig. 1:**
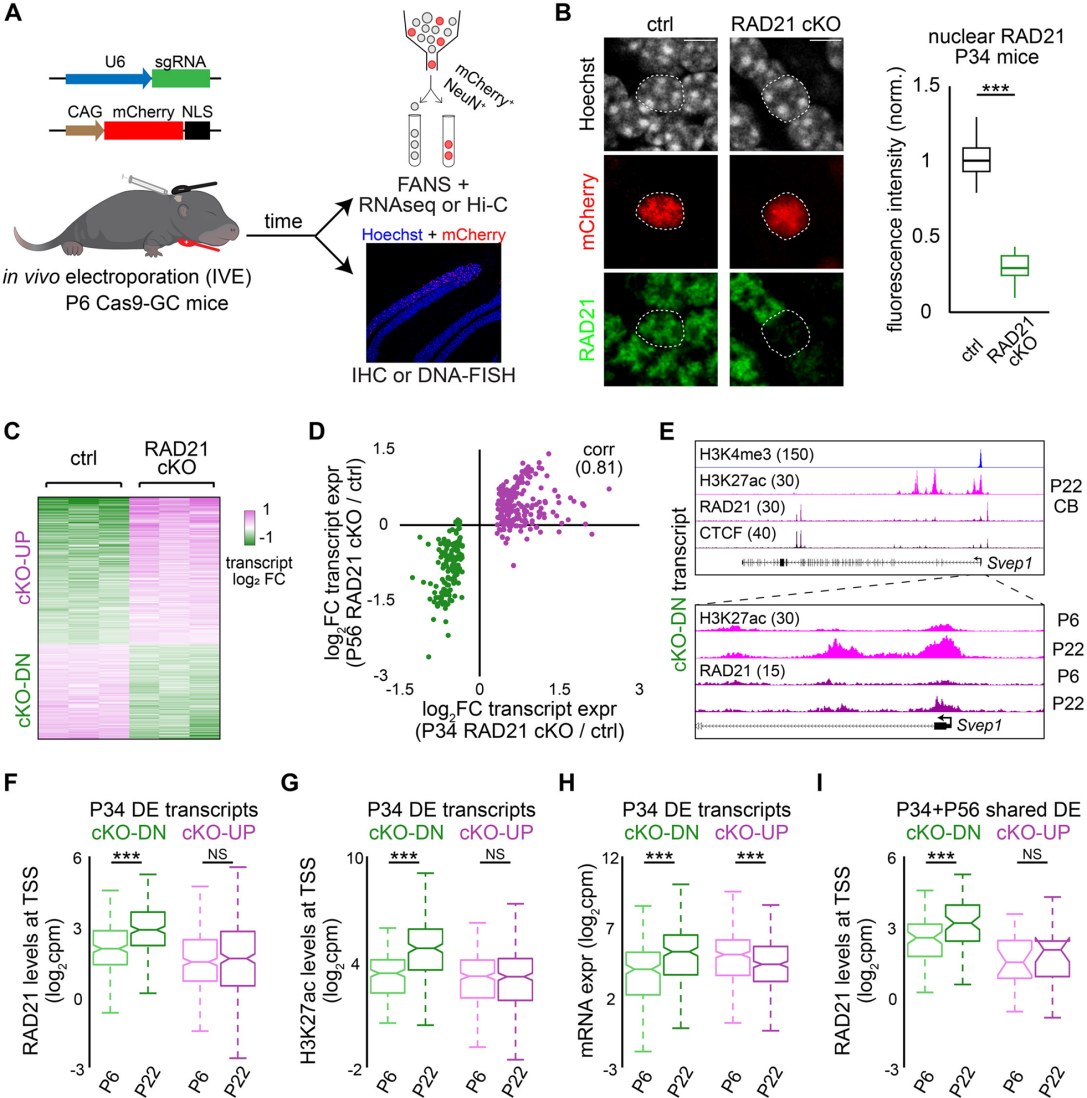
RAD21 depletion induces transcriptional changes in cerebellar granule neurons. **A** Postnatal day 6 (P6) mouse pups expressing Cas9 in granule neurons in the cerebellum (Cas9-GC) subjected to in vivo electroporation (IVE, left) using vectors encoding small guide RNA (sgRNA) targeting *Rad21* or a control sgRNA, together with the mCherry expression plasmid (left). Two to four weeks later, dissociated cerebellar granule neurons were immunolabeled with mCherry and NeuN antibodies to identify electroporated granule neurons, isolated with fluorescence-activated nuclei sorting (FANS), and subjected to RNA-seq or Hi-C analyses (right, top). Alternatively, cerebellar sections were subjected to immunohistochemistry or DNA fluorescence in situ hybridization (DNA-FISH) analyses (right, bottom). **B** Left, images of control or RAD21 conditional knockout (cKO) granule neurons from postnatal day 34 (P34) electroporated animals, labeled with antibodies against RAD21 and mCherry. The dashed line indicates the boundary of a nucleus, scale bar: 5 µm. Right, quantification of RAD21 immunofluorescence in mCherry-positive electroporated neurons, normalized to surrounding non-electroporated neurons. sgRNA targeting *Rad21* reduced RAD21 protein levels compared to control sgRNA in P34 mice (***P < 0.001 using unpaired two-tailed Student’s *t*-test, n = 30–32 mCherry-positive neurons). **C** Heatmap of differentially expressed genes upon RAD21 conditional knockout in P34 mice (FDR < 0.01, two-sided *P* value from negative binomial distribution with Benjamini–Hochberg post hoc test,* n* = 3 biological replicates). **D** Comparison of differentially expressed genes upon RAD21 depletion at P34 with our previous RNA-seq analyses from P56 mice [24]. The correlation coefficient (corr) is indicated. **E** Top, UCSC genome browser tracks showing H3K4me3, H3K27ac, RAD21, and CTCF ChIP-seq levels at the *Svep1* gene locus, whose expression is downregulated upon RAD21 conditional knockout. Bottom, a zoomed-in view of the *Svep1* promoter in P6 or P22 mouse cerebellum. H3K27ac and RAD21 levels at the *Svep1* gene promoter increased with cerebellar development. **F** and **G** RAD21 or H3K27ac levels at transcription start sites (TSS) in P6 or P22 cerebellum for RAD21 cKO-downregulated or upregulated transcripts as in (**C**). RAD21 or H3K27ac levels at the TSSs of RAD21 cKO-downregulated transcripts increased with development (***P < 0.001 using sign test, n = 211, 317 TSSs for cKO-DN, cKO-UP). **H** mRNA levels in P6 or P22 cerebellum for RAD21 cKO-downregulated or upregulated transcripts as in (**C**). RAD21 cKO-downregulated transcripts were increased during development, whereas RAD21 cKO-upregulated transcripts were decreased during development (***P < 0.001 using sign test, n = 211, 317 transcripts for cKO-DN, cKO-UP). (I) RAD21 levels at TSSs in P6 or P22 cerebellum for RAD21 cKO-downregulated or upregulated transcripts shared between the P34 and P56 RNA-seq datasets. RAD21 levels at the TSSs of shared RAD21 cKO-downregulated transcripts increased with development (***P < 0.001 using sign test, n = 71, 21 TSSs for cKO-DN, cKO-UP). Box plots in (**B**) and (**F**–**I**) show median, quartiles (box), and range (whiskers)

To investigate the roles of the cohesin complex in mature neurons, we induced the conditional knockout of the essential cohesin subunit RAD21 specifically in mouse cerebellar granule neurons after their initial developmental period in vivo. We achieved this by crossing LSL-Cas9 mice, which conditionally express the Cas9 endonuclease, with a transgenic mouse line where Cre recombinase expression is driven by the *Gabra6* gene promoter in mature granule neurons (Cas9-GC) during the second postnatal week [28]. We electroporated postnatal day 6 (P6) Cas9-GC mouse pups with a plasmid encoding a small guide RNA (sgRNA) targeting *Rad21* or a control genomic region together with a plasmid encoding a fluorescent protein mCherry-NLS, which localizes to the nucleus (Figs. 1A, B). Immunofluorescence analyses of the cerebellar cortex from P20 or P34 mice, at two or four weeks after electroporation, revealed reduced RAD21 expression in mCherry-labeled, RAD21 conditional knockout (cKO) granule neurons compared to control neurons, with more pronounced effects observed in P34 animals (Figs. 1B and S1A). Next, to assess RAD21’s role in gene expression, we performed RNA-seq analyses on control or RAD21 conditional knockout granule neurons isolated from P34 mice using fluorescence-activated nuclei sorting (FANS) (Fig. 1A). We found that RAD21 depletion induced both gene upregulation and downregulation in P34 animals, whereas little or no changes were observed at P20 (Figs. 1C and S1B), suggesting that near-complete loss of RAD21 was required to achieve measurable effects on transcription. In other analyses, the selective knockout of RAD21 in adult mice led to similar gene expression changes in P56 animals [24] as those observed in P34 animals, with genes downregulated upon RAD21 depletion in both P34 and P56 mice encoding cell membrane proteins or factors involved in differentiation (Figs. 1D and S1C; Table S1). These findings indicate that RAD21 controls gene expression in mature granule neurons.

To determine how these genes are regulated in granule neurons, we examined their genomic enrichment for RAD21 and CTCF, as well as histone modifications associated with transcriptional activation, such as H3K4 trimethylation (H3K4me3) and H3K27 acetylation (H3K27ac). We performed ChIP-seq analyses at postnatal day 6 (P6), when the majority of the cerebellum comprises granule cell precursors and immature post-mitotic granule neurons, and at postnatal day 22 (P22), when granule neurons have matured and integrated into neural circuits [31]. Granule neurons constitute > 85% of cerebellar cells throughout these developmental stages [35]. At the *Svep1* gene, which is downregulated upon RAD21 depletion, we observed that RAD21 enrichment and H3K27ac levels near its transcription start site (TSS) increased with cerebellar development (Fig. 1E). These findings extended to the entire group of RAD21 cKO-downregulated genes, which showed increased RAD21 recruitment and H3K27ac deposition near their TSSs, along with concomitant increases in gene expression during development (Figs. 1F–H and S1D-F). Similar RAD21 binding patterns were observed at the TSSs of the shared group of RAD21 cKO-downregulated genes in P34 and P56 animals (Figs. 1I and S1G), indicating that ongoing cohesin recruitment beyond development is required to maintain their expression. In contrast, TSSs of RAD21 cKO-upregulated genes showed little or no developmental changes in RAD21 or H3K27ac levels (Figs. 1F, G and S1D, E). Together, these results suggest that the cohesin complex directly regulates target genes to maintain their expression in mature granule neurons.

### Cohesin promotes local genomic interactions at its target genes

**Fig. 2:**
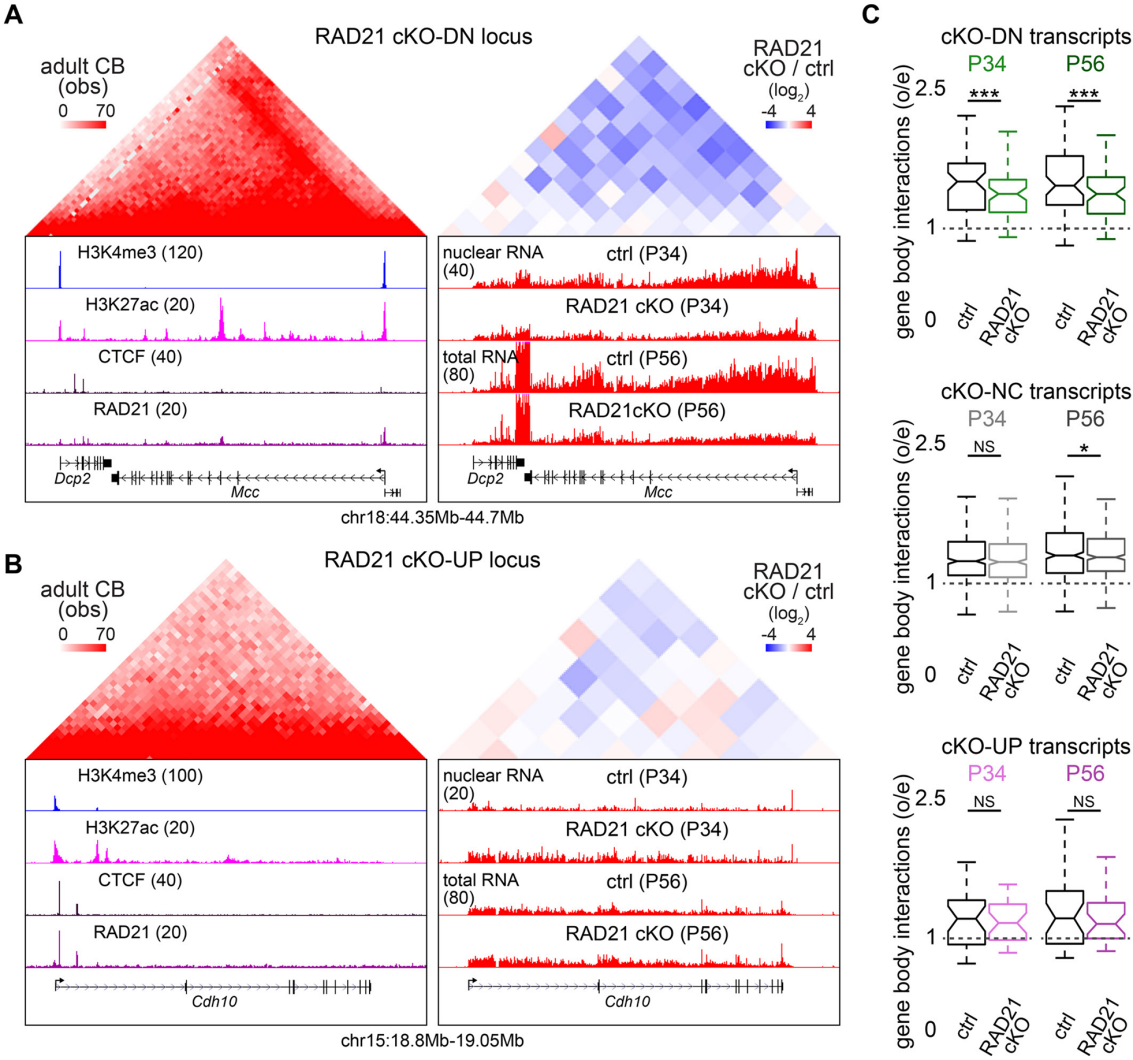
Cohesin promotes local genomic interactions at its target genes. **A**, **B** Left, Hi-C map of the *Mcc* (**A**) or *Cdh10* (**B**) locus aligned with UCSC genome browser tracks showing H3K4me3, H3K27ac, CTCF, and RAD21 ChIP-seq levels in P22 cerebellum. Right, fold changes in local genomic interactions between RAD21 conditional knockout and the control condition (top) aligned with RNA-seq tracks from the control condition and upon RAD21 depletion in P34 or P56 animals (bottom). RAD21 depletion reduced local genomic interactions within the *Mcc* gene body and reduced *Mcc* expression, whereas interactions within *Cdh10* locus showed only modest changes despite an increase in *Cdh10* expression. **C** Quantification of local gene body interactions in the control condition or upon RAD21 conditional knockout in P34 or P56 mice for transcripts that were RAD21 cKO-downregulated (DN, top), not changed (NC, middle), or upregulated (UP, bottom). RAD21 depletion robustly reduced local gene body interactions at transcriptionally downregulated genes (***P < 0.0001, *P < 0.01 using sign test, n = 67, 371, 30 for shared cKO-DN, shared cKO-NC, shared cKO-UP). Box plots in (**C**) show median, quartiles (box), and range (whiskers)

We next asked whether the effects of RAD21 on gene expression might be associated with its regulation of local genomic interactions in mature granule neurons. Using a deeply sequenced Hi-C contact map of adult cerebellum [24, 31], we visualized the *Mcc* gene locus, whose expression was downregulated upon RAD21 depletion, and observed robust genomic interactions between the H3K4me3-marked *Mcc* gene promoter and its H3K27ac-marked intragenic enhancers (Fig. 2A). Notably, RAD21 occupied the *Mcc* promoter and a subset of its enhancers. To test cohesin’s functions at this gene locus, we performed FANS-Hi-C analyses of P34 RAD21 conditional knockout or control granule neurons and integrated these data with our previous Hi-C datasets from P56 animals [24]. We found that RAD21 depletion reduced local interactions within the *Mcc* gene locus, including those between the *Mcc* promoter and its enhancers (Fig. 2A, right). In contrast, the *Cdh10* gene locus, whose expression was upregulated upon RAD21 depletion, exhibited weak local interactions in the adult cerebellum, and these interactions showed little or no changes following RAD21 conditional knockout in granule neurons (Fig. 2B). These results suggest that cohesin selectively promotes local genomic interactions at certain genes such as *Mcc*.

To determine whether cohesin’s roles at the *Mcc* and *Cdh10* loci extended genome-wide, we quantified genomic interactions along the bodies of genes regulated by RAD21. We found that RAD21 depletion robustly reduced intragenic interactions at RAD21 cKO-downregulated genes, while RAD21 cKO-upregulated genes showed little or no changes (Figs. 2C and S2). Moreover, in control granule neurons, RAD21 cKO-downregulated genes exhibited stronger gene body interactions than cKO-upregulated genes, which is consistent with their higher expression in mature granule neurons (Figs. 1H and 2C). These results indicate that cohesin promotes intragenic interactions at its target genes in mature granule neurons.

**Fig. 3:**
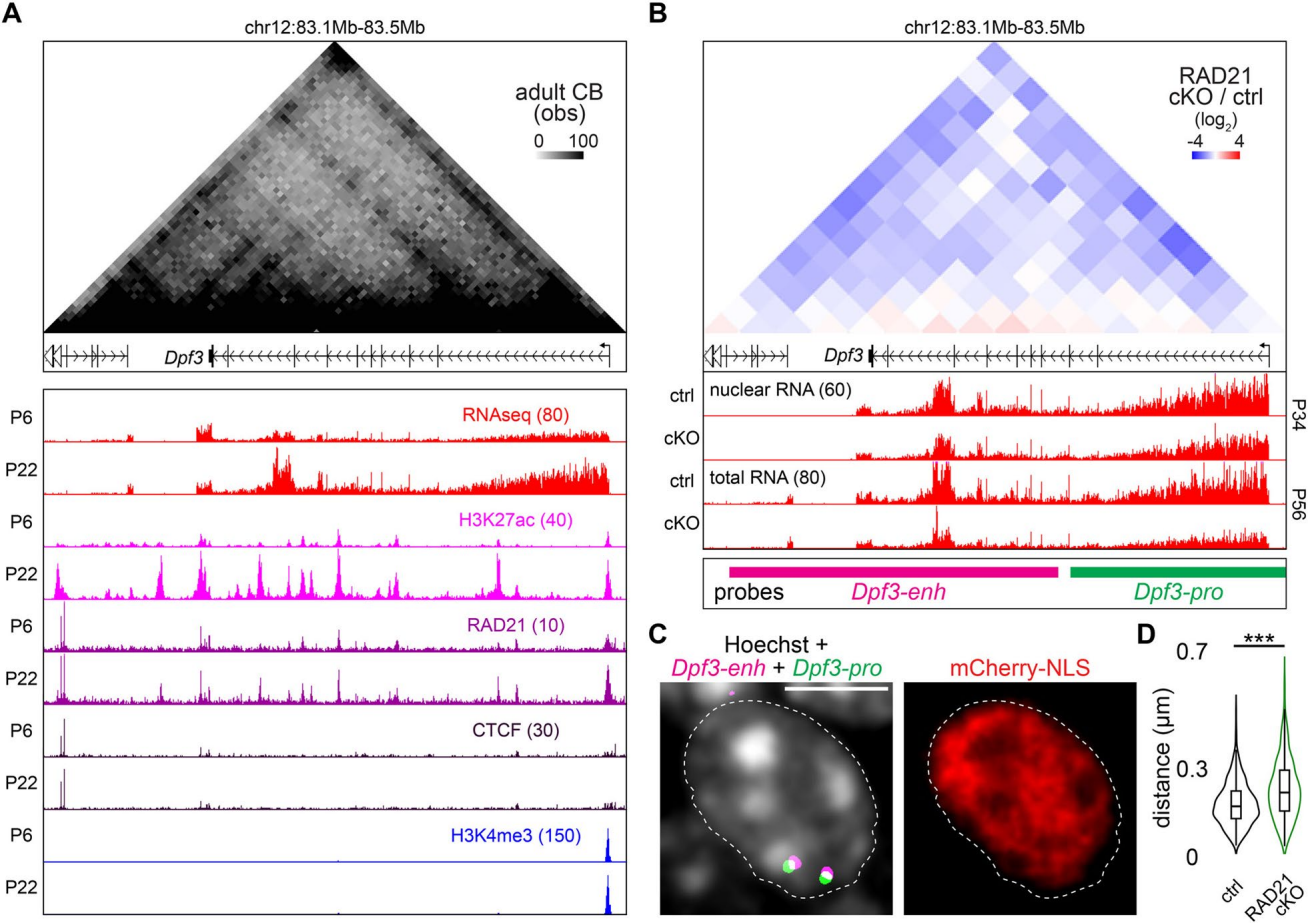
Cohesin maintains spatial proximity between the *Dpf3* promoter and its enhancers and promotes *Dpf3* expression. **A** UCSC genome browser tracks of mRNA, H3K27ac, RAD21, CTCF, and H3K4me3 levels at the *Dpf3* locus in P6 and P22 cerebellum aligned to the Hi-C contact map of this locus in the adult cerebellum. The *Dpf3* gene was transcriptionally upregulated during development and exhibited robust local genomic interactions in the adult cerebellum. **B** Top, fold changes in local genomic interactions at the *Dpf3* gene locus upon RAD21 conditional knockout compared to the control condition together with UCSC genome browser tracks of RNA levels in P34 or P56 cerebellum. Local genomic interactions at the *Dpf3* locus and *Dpf3* expression were reduced upon RAD21 depletion. Bottom, *Dpf3* enhancers and promoter targeted using DNA FISH probes. **C** Images of granule neurons electroporated with a plasmid encoding the fluorescence protein mCherry-NLS and labeled with DNA FISH probes targeting the *Dpf3* promoter (green) and enhancers (magenta) together with an mCherry antibody (red) and the Hoechst DNA dye (white). The dashed line indicates the boundary of a nucleus, scale bar: 5 µm. **D** 3D distances between the *Dpf3* promoter and enhancers in the control condition or upon RAD21 conditional knockout. The spatial distance between the *Dpf3* promoter and its enhancers increased upon RAD21 depletion (***P < 0.001, Kruskal–Wallis test with Dunn’s post hoc test,* n* = 576, 596 loci for control, RAD21 cKO). Box plots in (**D**) show median, quartiles (box), and range (whiskers)

We next assessed how cohesin’s regulation of local genomic organization affects the spatial clustering of these regions in granule neurons. We focused on the *Dpf3* gene locus, whose enhancers showed increases in H3K27ac deposition and RAD21 binding during development, alongside the upregulation of *Dpf3* expression (Fig. 3A). In the adult cerebellum, the *Dpf3* gene locus exhibited robust local interactions, including between its promoter and enhancers (Fig. 3A). RAD21 depletion reduced these local genomic interactions and also reduced *Dpf3* expression (Fig. 3B). We then performed three-dimensional (3D) DNA fluorescence in situ hybridization (FISH) analyses using probes targeting the *Dpf3* promoter or enhancers to visualize their spatial proximity in RAD21 conditional knockout or control granule neurons (Figs. 3B, C). We found that the 3D distance between the *Dpf3* promoter and its enhancers increased upon RAD21 depletion (Fig. 3D), consistent with their weakened local interactions. These findings suggest that RAD21 brings target gene promoters and enhancers into close spatial proximity to activate transcription.

### Cohesin maintains distal intergenic enhancers within the A compartment

**Fig. 4:**
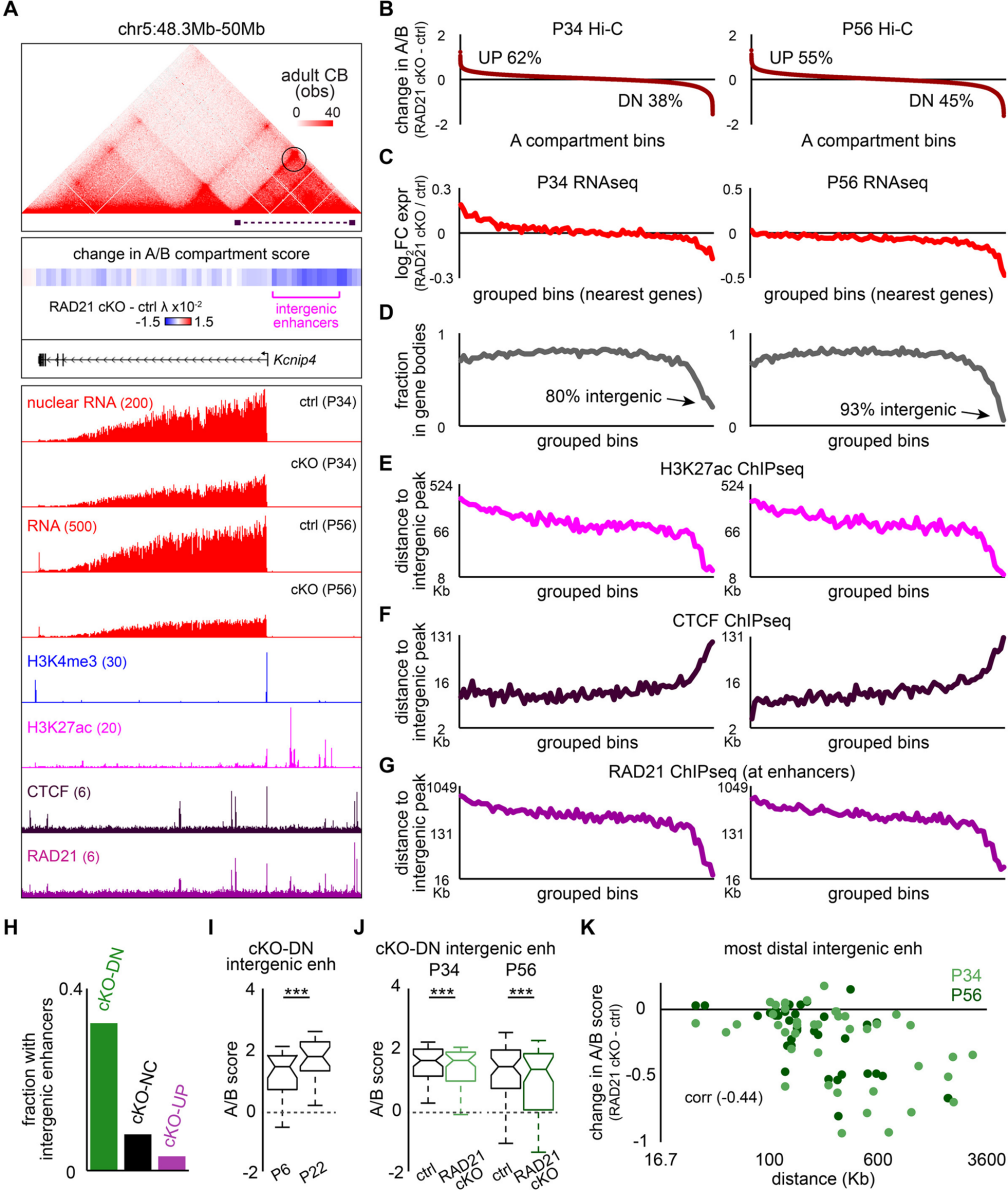
Cohesin maintains distal intergenic enhancers of target genes in the A compartment. **A** Top, Hi-C contact map of the *Kcnip4* locus in adult cerebellum. The purple dashed line indicates a topological domain encompassing the *Kcnip4* promoter and intergenic enhancers. The chromatin loop formed between the domain boundaries is circled. Middle, change in A/B compartment score across the *Kcnip4* locus upon RAD21 conditional knockout compared to the control condition in P56 cerebellum. Bottom, UCSC genome browser tracks of *Kcnip4* RNA levels in the control condition or upon RAD21 conditional knockout in P34 or P56 cerebellum, alongside H3K4me3, H3K27ac, CTCF, and RAD21 levels at the *Kcnip4* locus in P22 cerebellum. Intergenic enhancers extending up to 330 Kb upstream of the *Kcnip4* gene show reduced association with the A compartment upon RAD21 depletion, concomitant with decreased *Kcnip4* expression. **B** Changes in A/B compartment scores for all A compartment bins at 25 Kb resolution upon RAD21 conditional knockout compared to the control condition in P34 (left) or P56 (right) cerebellum, with bins sorted by score. **C**, **D** For groups of bins sorted as in (**B**), the average fold change in expression of the nearest gene upon RAD21 conditional knockout compared to the control condition (**C**) or the fraction of bins overlapping with expressed genes (**D**) in P34 (left) or P56 (right) cerebellum. Regions with reduced A/B compartment scores following RAD21 depletion were located near downregulated genes, but were mostly intergenic. **E–G** For groups of bins sorted as in (**B**), the average distance to the nearest intergenic H3K27ac peak (**E**), CTCF peak (**F**), or RAD21 enhancer peak (**G**). Regions with reduced A/B compartment scores were located near active H3K27ac-marked enhancers bound by RAD21, but not by CTCF. (H) Fraction of RAD21 cKO-downregulated (DN), not changed (NC), or upregulated (UP) transcripts that were located adjacent to intergenic H3K27ac-marked enhancers. **I** A/B compartment score of intergenic enhancers adjacent to shared RAD21 cKO-downregulated genes as in Fig. 1I, in P6 or P22 cerebellum. These intergenic enhancers increased genomic interactions with the A compartment during cerebellar development (***P < 0.0001 using sign test, n = 22 loci). **J** A/B compartment score of intergenic enhancers adjacent to RAD21 cKO-downregulated genes in the control condition or upon RAD21 conditional knockout in P34 or P56 cerebellum. These intergenic enhancers exhibit decreased genomic interactions with the A compartment upon RAD21 depletion (***P < 0.0001 using sign test, n = 52, 48 loci for P34, P56). **K** Correlation between the change in the A/B compartment score of intergenic enhancers upon RAD21 conditional knockout and the genomic distance from RAD21 cKO-downregulated genes to their most distal intergenic enhancers in P34 or P56 cerebellum. Following RAD21 depletion, distal intergenic enhancers located hundreds of kilobases from downregulated genes showed greater reductions in A compartment interactions compared to more proximal enhancers (*P* < 0.0001, t-test for correlation, n = 100 loci). Box plots in (**I**) and (**J**) show median, quartiles (box), and range (whiskers)

Having identified a function for cohesin in promoting local genomic interactions at its target genes, we investigated whether it has other roles in genome organization linked to gene transcription such as the partitioning of regions within the A or B compartment. At the *Kcnip4* gene locus, whose expression depends on cohesin, we observed that the *Kcnip4* gene promoter formed interactions with upstream intergenic enhancers marked by H3K27ac and bound by RAD21 (Figs. 4A and S3A). To determine the effects of RAD21 depletion on compartmentalization, we examined the A/B compartment scores at this locus and found that *Kcnip4*’s distal intergenic enhancers shifted from the transcriptionally active A compartment toward the B compartment upon RAD21 depletion (Figs. 4A and S3B). This result suggests that cohesin maintains these enhancer regions within the transcriptionally active compartment in mature granule neurons.

Given the potential link between cohesin and compartmentalization at the *Kcnip4* gene locus, we examined this relationship for all transcriptionally active regions in the genome. We identified genomic regions with increased and decreased association with the A compartment upon RAD21 depletion and assessed the genomic features that were enriched at these regions (Figs. 4B and S3C). Consistent with our findings at the *Kcnip4* gene locus, a small number of regions showing strong reductions in A compartment association were located near genes downregulated upon RAD21 depletion in granule neurons (Figs. 4C and S3D). Interestingly, these regions were also predominantly intergenic (Figs. 4D and S3E), indicating that these pronounced compartment changes did not occur within the bodies of RAD21 cKO-downregulated genes. We therefore determined whether these intergenic regions contained active, H3K27ac-marked enhancers or CTCF-bound sites which often act as insulator elements. We found that intergenic regions with reduced A compartment association were enriched for H3K27ac-marked, RAD21-bound enhancers, but depleted for CTCF binding (Figs. 4E–G and S3F, G). These results suggest that RAD21 maintains intergenic enhancers within the transcriptionally active A compartment in granule neurons.

We further investigated cohesin’s relationship with intergenic enhancers. We found that a higher proportion of RAD21 cKO-downregulated genes were positioned nearby active intergenic enhancers compared with RAD21 cKO-upregulated or unchanged genes (Fig. 4H). These intergenic enhancers near RAD21 cKO-downregulated genes exhibited stronger association with the A compartment in mature granule neurons, and this association was impaired upon RAD21 depletion (Figs. 4I, J and S3H). Because cohesin's role in gene regulation depends on the genomic distance between gene promoters and their enhancers [22], we next examined whether the intergenic enhancer distance from genes correlated with its change in A compartment association. Strikingly, we found that distal intergenic enhancers located more than 100 Kb away from RAD21 target genes showed robust reductions in A compartment association upon RAD21 depletion, whereas more proximal intergenic enhancers retained this association (Figs. 4A, K, S3A, B, and S4). These results indicate that cohesin is required to retain distal intergenic enhancers within the transcriptionally active A compartment. Together, our findings reveal that cohesin maintains both local promoter-enhancer interactions and the compartmentalization of distal intergenic enhancers, and these mechanisms are tightly linked to its control of target gene expression in mature granule neurons.

## Discussion

In our study, we uncover roles for the cohesin complex in genome organization across different length scales to regulate gene expression in the mouse cerebellum in vivo. Notably, we find that both local genomic interactions and genome compartmentalization are sensitive to cohesin loss in granule neurons during late cerebellar development and adulthood. Moreover, cohesin is required for the transcription of genes upregulated during development and that remain active in mature granule neurons, underscoring the important roles for genome organization in the adult brain, including in learning and memory [24].

An established role for cohesin is to increase the probability of forming promoter-enhancer contacts at its target genes by bringing their promoters into close 3D proximity with regulatory enhancers. This spatial clustering concentrates transcriptional machinery at enhancers and gene promoters [36, 37]. Recent studies also show that cohesin regulates gene expression by facilitating promoter-enhancer interactions at distances over 50 Kb, while at shorter distances gene expression does not appear to require loop extrusion [22, 37–39]. In granule neurons, we observe that more than one-third of cohesin’s target genes are located next to intergenic enhancers, and a sizeable number of these are positioned more than 100 Kb away. Interestingly, we find that cohesin not only brings enhancers into close proximity to target gene promoters, but also promotes the compartmentalization of distal intergenic enhancers. Given that enhancers, including distal ones, typically reside in the A compartment in mammalian cells [40], our data suggest that cohesin helps tether distal intergenic enhancers to this transcriptionally active compartment. These findings extend cohesin’s roles in both promoting and restricting chromatin compartment interactions in cells [13, 21]. Collectively, cohesin establishes an active microenvironment enriched for gene promoter and enhancer elements that supports proper gene transcription in the cerebellum.

We also broaden our understanding of cohesin’s in vivo functions beyond neuronal development. Although we focused on RAD21, which bridges SMC1 and SMC3 within the cohesin ring, the loss of any core cohesin subunit can compromise complex integrity and therefore disrupt chromatin looping [41]. Genetic mutations in SMC1, SMC3, RAD21, and SCC3 have been implicated in neurodevelopmental disorders such as Cornelia de Lange syndrome (CdLS) [42], but the subunit-specific contributions to gene regulation, particularly in the adult brain, remains unclear. Additionally, defects in chromatin looping may be associated with human neurological disorders and cancer in adults [43–45]. Our work provides a foundation for studying genome organization in mature neurons in the brain and its dysregulation in disease models.

## Conclusions

We conditionally deleted the core cohesin subunit RAD21 and found that cohesin is essential for gene expression in mature cerebellar granule neurons. At target genes, RAD21 binds to promoters and enhancers to strengthen their genomic interactions and recruits distal intergenic enhancers to the transcriptionally active A compartment. Collectively, these findings advance our understanding of cohesin’s roles in genome organization and transcriptional regulation in the adult brain in vivo.

## Supplementary Information

Additional file 1 (PDF 2280 KB)

Additional file 2 (XLSX 143 KB)

## Data Availability

Raw and processed sequencing data generated for this study are available from 4DN data portal (https://data.4dnucleome.org/) deposited under Tomoko Yamada, NW.

## Abbreviations

3D: Three-dimensional
Kb: Kilobases
Mb: Megabases
TSS: Transcription start site
cKO: Conditional knockout
Cas9-GC: Conditional expression of Cas9 in mature cerebellar granule neurons
CTCF: CCCTC-binding factor
TAD: Topologically associating domain
sgRNA: Single guide RNA
CRISPR: Clustered regularly interspaced short palindromic repeats
FANS: Fluorescence activated nuclei sorting
Hi-C: High-throughput chromosome conformation capture
DNA FISH: DNA fluorescence in situ hybridization
