# Supplementary material for: Cohesin regulation of genome organization in mature granule neurons in the mouse cerebellum
Source: Epigenetics Chromatin. 2025 Sep 26;18:60. doi: 10.1186/s13072-025-00625-2 (PMC12465447; doi:10.1186/s13072-025-00625-2)
Supplement: Supplementary file 1 — Additional file 1 (PDF 2280 KB) [file 13072_2025_625_MOESM1_ESM.pdf]

Supplementary Figure Legends

Figure S1 Characterization of genes dysregulated upon RAD21 depletion in mature neurons

(A) Quantification of RAD21 immunofluorescence in mCherry-positive electroporated neurons from postnatal day 20 (P20) animals as in Fig. 1B (***P < 0.001 using unpaired two-tailed Student's t-test, n = 31 mCherry-positive neurons). (B) Fold changes in expression of the transcripts from Fig. 1C in P20 or P34 granule neurons following RAD21 conditional knockout (cKO). Differentially expressed (DE) transcripts in P34 mice show only mild changes at P20. (C) Annotation of downregulated or upregulated genes upon RAD21 conditional knockout using DAVID. (D-G) Fold changes in RAD21 levels (D, G), H3K27ac levels (E), or mRNA expression (F) between P6 and P22 cerebellum at regions downregulated or upregulated upon RAD21 conditional knockout as in Figs. 1F-I.

Box plots in (A), (B), and (D-G) show median, quartiles (box), and range (whiskers).

Figure S2 Effects of RAD21 depletion on gene body interactions

Fold changes in gene body interactions at transcripts downregulated (DN), not changed (NC), or upregulated (UP) upon RAD21 conditional knockout in P34 or P56 cerebellum as in Fig. 2C. Box plots show median, quartiles (box), and range (whiskers).

Figure S3 RAD21 depletion disrupts the association of distal intergenic enhancers with the A compartment

(A) Hi-C contact maps of the *Kcnip4* locus in control or RAD21 conditional knockout cerebellum, as in Fig. 4A. The topological domain encompassing the *Kcnip4* promoter and intergenic enhancers is indicated by the highlighted dashed line, and the chromatin loop formed between the

domain boundaries is circled. (B) Top, Hi-C contact maps showing interactions between the topological domain from (A) and a ~15 Mb region containing this topological domain and a neighboring B compartment region (denoted with the green line), in control or RAD21 conditional knockout cerebellum. Bottom, A/B compartment scores along this region, with the topological domain from (A) highlighted in yellow. (C) Changes in A/B compartment scores at 25 Kb resolution for each biological replicate from P34 or P56 cerebellum. A compartment bins are from Fig. 4B in the same rank order. (D-G) Fold change in mRNA expression of nearby genes (D), fraction overlapping with expressed genes (E), or distance to the nearest intergenic H3K27ac or CTCF peak (F, G) for regions with decreased (DN, $\Delta < -1$), unchanged (NC, $|\Delta| < 0.05$), or increased (UP, $\Delta > 0.5$) A/B compartment scores upon RAD21 conditional knockout in P34 or P56 cerebellum, as in Figs. 4C-F (*FDR < 0.01, **FDR < 0.001, ***FDR < 0.0001, Mood's median test versus the unchanged group with Benjamini-Hochberg correction). (H) Changes in A/B compartment scores at intergenic enhancers adjacent to genes downregulated upon RAD21 conditional knockout in P34 or P56 cerebellum as in Fig. 4J.

Box plots (D) and (F-H) show median, quartiles (box), and range (whiskers).

Figure S4 RAD21 depletion has little or no effects on the compartmentalization of proximal intergenic enhancers

(A) Top, Hi-C contact map of the *Ndrg3* locus in adult cerebellum. The highlighted dashed line indicates a topological domain encompassing the *Ndrg3* promoter and proximal intergenic enhancers. The chromatin loop formed between the domain boundaries is circled. Bottom, UCSC genome browser tracks of H3K4me3, H3K27ac, CTCF, and RAD21 levels at the *Ndrg3* locus in P22 cerebellum. (B) Hi-C contact maps of the *Ndrg3* locus in control or RAD21 conditional

knockout cerebellum, as in (A). Local genomic interactions at this locus are weakened upon RAD21 depletion. (C) Top, Hi-C contact maps showing interactions between the topological domain from (A) and a ~10 Mb region containing this topological domain and a neighboring B compartment region (denoted with the green line), in control or RAD21 conditional knockout cerebellum. Bottom, A/B compartment scores along this region, with the topological domain from (A) highlighted in yellow. (D, E) Left, fold changes in mRNA expression for *Ndrg3* (D) or *Kcnip4* (E) upon RAD21 conditional knockout. Right, changes in A/B compartment scores for intergenic enhancers near these genes. Both *Ndrg3* and *Kcnip4* showed reduced expression following RAD21 depletion. However, while distal intergenic enhancers up to 330 Kb away from *Kcnip4* showed reduced association with the A compartment after RAD21 depletion, as in Fig. 4A, proximal intergenic enhancers within 50 Kb of *Ndrg3* showed no changes in A/B scores in P34 or P56 cerebellum. Data show mean and error bars denote s.e.m.

Supplementary Table

Table S1. Differentially expressed transcripts in P34 and P56 cerebellum following RAD21 conditional knockout in granule neurons

Figure S1

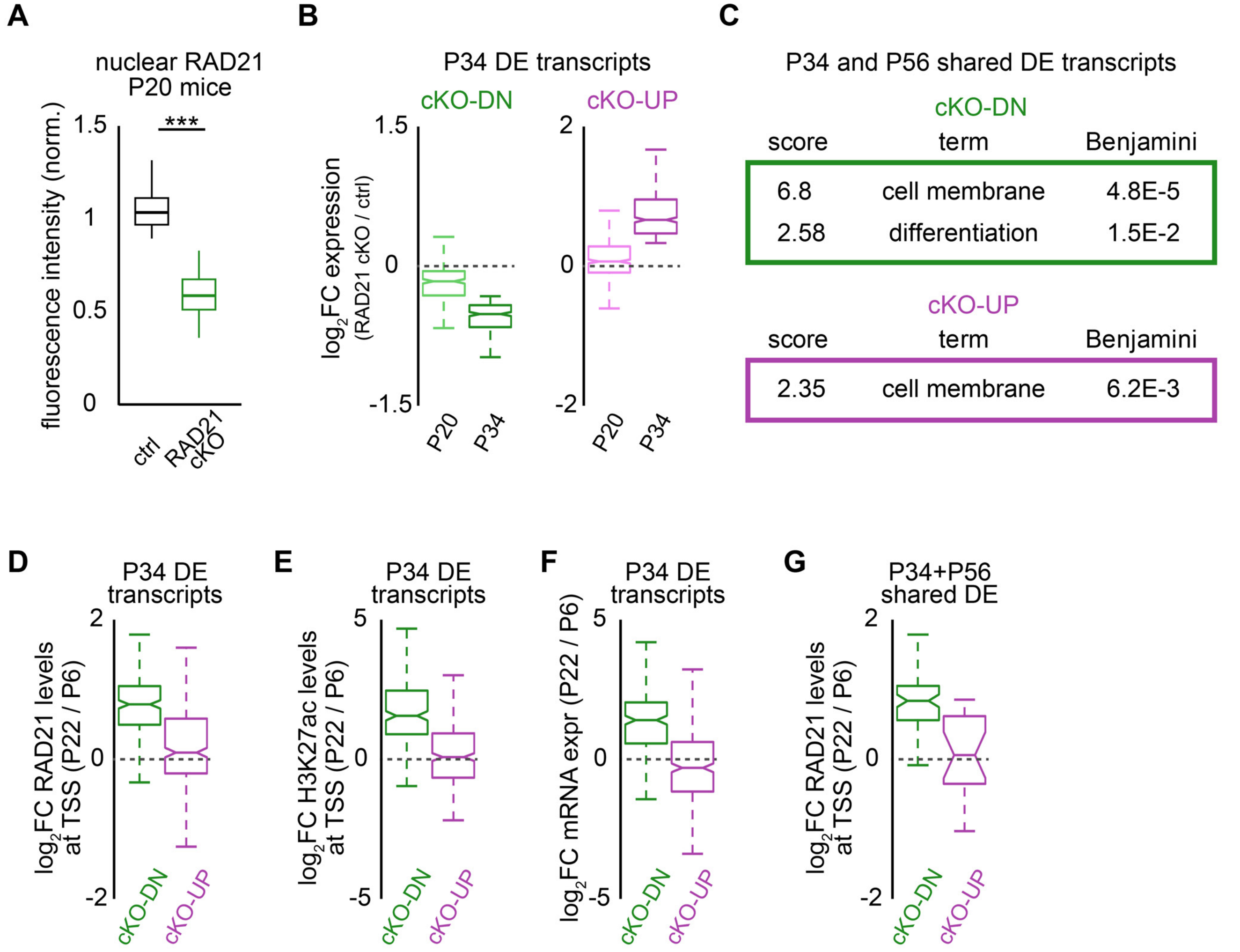

A
nuclear RAD21
P20 mice
fluorescence intensity (norm.)
***
1.5
1
0.5
0
ctrl
RAD21 cKO
B
P34 DE transcripts
cKO-DN
cKO-UP
log2FC expression
(RAD21 cKO / ctrl)
1.5
0
-1.5
2
0
-2
P20
P34
P20
P34
C
P34 and P56 shared DE transcripts
cKO-DN
score
term
Benjamini
6.8
cell membrane
4.8E-5
2.58
differentiation
1.5E-2
cKO-UP
score
term
Benjamini
2.35
cell membrane
6.2E-3
D
P34 DE transcripts
log2FC RAD21 levels at TSS (P22 / P6)
2
0
-2
cKO-DN
cKO-UP
E
P34 DE transcripts
log2FC H3K27ac levels at TSS (P22 / P6)
5
0
-5
cKO-DN
cKO-UP
F
P34 DE transcripts
log2FC mRNA expr (P22 / P6)
5
0
-5
cKO-DN
cKO-UP
G
P34+P56 shared DE
log2FC RAD21 levels at TSS (P22 / P6)
2
0
-2
cKO-DN
cKO-UP

Figure S2

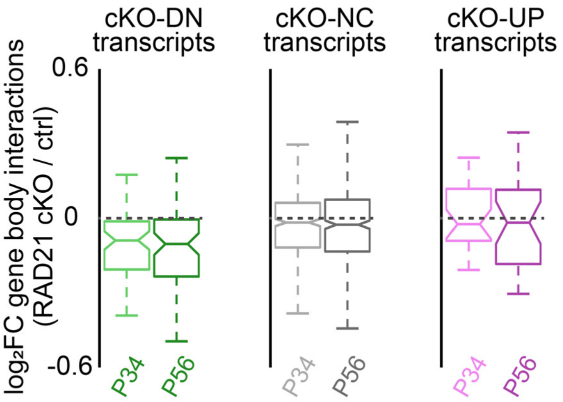

cKO-DN transcripts
cKO-NC transcripts
cKO-UP transcripts
log₂FC gene body interactions (RAD21 cKO / ctrl)
0.6
0
-0.6
P34
P56
P34
P56
P34
P56

Figure S3

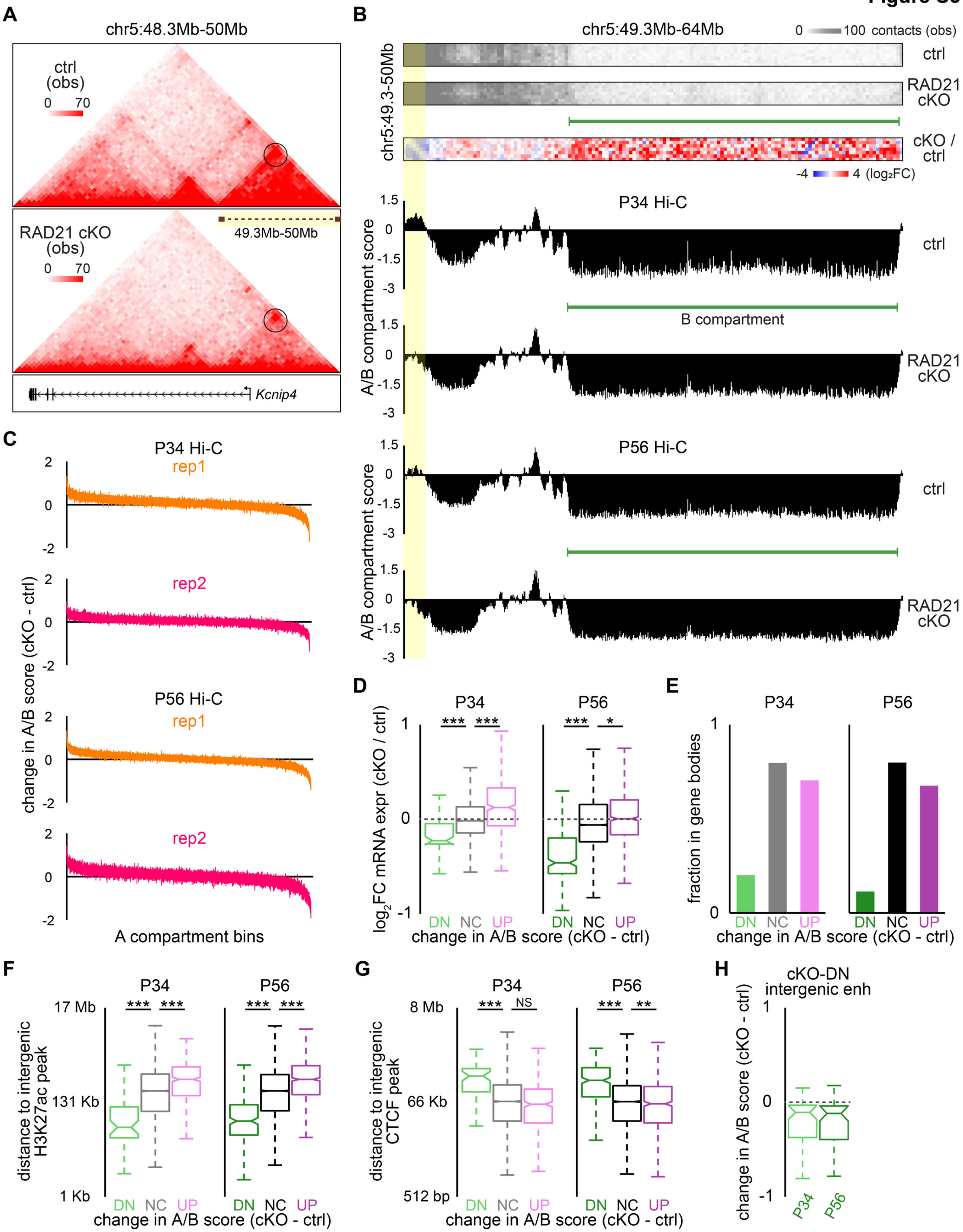

A
chr5:48.3Mb-50Mb
ctrl (obs)
0 70
RAD21 cKO (obs)
0 70
49.3Mb-50Mb
Kcnip4
B
chr5:49.3Mb-64Mb
0 100 contacts (obs)
chr5:49.3-50Mb
ctrl
RAD21 cKO
cKO / ctrl
-4 4 (log2FC)
P34 Hi-C
A/B compartment score
1.5
0
-1.5
-3
B compartment
P56 Hi-C
C
P34 Hi-C
rep1
rep2
P56 Hi-C
change in A/B score (cKO - ctrl)
A compartment bins
D
P34
P56
log2FC mRNA expr (cKO / ctrl)
DN
NC
UP
change in A/B score (cKO - ctrl)
E
fraction in gene bodies
F
distance to intergenic H3K27ac peak
17 Mb
131 Kb
1 Kb
G
distance to intergenic CTCF peak
8 Mb
66 Kb
512 bp
NS
H
cKO-DN intergenic enh

Figure S4

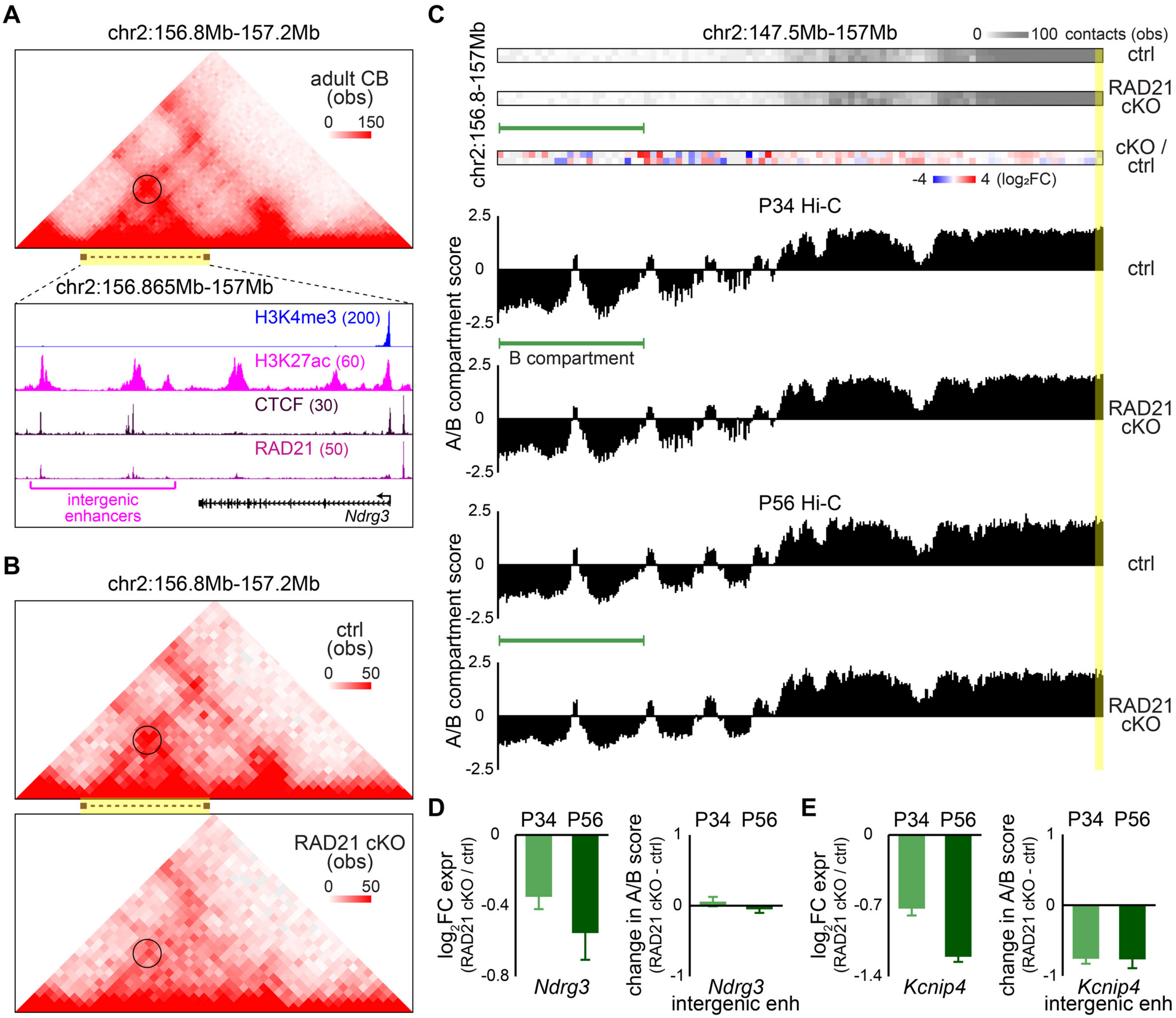
A
chr2:156.8Mb-157.2Mb
adult CB (obs)
0 150
chr2:156.865Mb-157Mb
H3K4me3 (200)
H3K27ac (60)
CTCF (30)
RAD21 (50)
intergenic enhancers
Ndrg3
B
chr2:156.8Mb-157.2Mb
ctrl (obs)
0 50
RAD21 cKO (obs)
0 50
C
chr2:147.5Mb-157Mb
0 100 contacts (obs)
chr2:156.8-157Mb
ctrl
RAD21 cKO
cKO / ctrl
-4 4 (log₂FC)
P34 Hi-C
A/B compartment score
2.5
0
-2.5
B compartment
ctrl
RAD21 cKO
P56 Hi-C
D
P34 P56
log2FC expr (RAD21 cKO / ctrl)
0
-0.4
-0.8
Ndrg3
change in A/B score (RAD21 cKO - ctrl)
1
0
-1
Ndrg3 intergenic enh
E
-0.7
-1.4
Kcnip4
Kcnip4 intergenic enh
